# A Phase I study of the novel immunomodulatory agent PG545 (pixatimod) in subjects with advanced solid tumours

**DOI:** 10.1038/s41416-018-0006-0

**Published:** 2018-03-13

**Authors:** Keith Dredge, Todd V. Brennan, Edward Hammond, Jason D. Lickliter, Liwen Lin, Darryn Bampton, Paul Handley, Fleur Lankesheer, Glynn Morrish, Yiping Yang, Michael P. Brown, Michael Millward

**Affiliations:** 1 Zucero Therapeutics, Brisbane, QLD Australia; 2 Department of Surgery, Duke University Medical Center, Durham, NC USA; 3 Nucleus Network, Melbourne, VIC Australia; 4 Progen Pharmaceuticals Ltd, Brisbane, QLD Australia; 5 Clinical Network Services, Brisbane, QLD Australia; 6 Departments of Medicine and Immunology, Duke University Medical Center, Durham, NC USA; 7 Cancer Clinical Trials Unit, Royal Adelaide Hospital; Centre for Cancer Biology, SA Pathology and University of South Australia; Discipline of Medicine, University of Adelaide, Adelaide, Australia; 8 Linear Clinical Research; Sir Charles Gairdner Hospital, University of Western Australia, WA Perth, Australia; 9 Present Address: School of Humanities and Social Science, The University of Newcastle, Newcastle, NSW Australia

**Keywords:** Drug development, Cancer immunotherapy

## Abstract

**Background:**

PG545 (pixatimod) is a novel immunomodulatory agent, which has been demonstrated to stimulate innate immune responses against tumours in preclinical cancer models.

**Methods:**

This Phase I study investigated the safety, tolerability, pharmacokinetics, pharmacodynamics and preliminary efficacy of PG545 monotherapy. Escalating doses of PG545 were administered to patients with advanced solid malignancies as a weekly 1-h intravenous infusion.

**Results:**

Twenty-three subjects were enrolled across four cohorts (25, 50, 100 and 150 mg). Three dose-limiting toxicities (DLTs)—hypertension (2), epistaxis (1)—occurred in the 150 mg cohort. No DLTs were noted in the 100 mg cohort, which was identified as the maximum-tolerated dose. No objective responses were reported. Best response was stable disease up to 24 weeks, with the disease control rate in evaluable subjects of 38%. Exposure was proportional up to 100 mg and mean half-life was 141 h. The pharmacodynamic data revealed increases in innate immune cell activation, plasma IFNγ, TNFα, IP-10 and MCP-1.

**Conclusion:**

PG545 demonstrated a tolerable safety profile, proportional PK, evidence of immune cell stimulation and disease control in some subjects. Taken together, these data support the proposed mechanism of action, which represents a promising approach for use in combination with existing therapies.

## Introduction

Immunotherapy is an effective treatment option for cancer patients across a broad range of tumour types. The durable and long-lasting anti-tumour activity observed in patients responding to immune checkpoint inhibitors have propelled multiple agents into mainstream cancer therapy.^1^ Nevertheless, most patients do not respond^2^ and intrinsic resistance is common,^3^ so finding therapeutic combinations that convert nonresponders to responders is critical to expand the utility of cancer immunotherapy.

Efforts are underway to characterize how the crosstalk between the innate and adaptive immune response influences sustained antigen-specific immunity.^4^ For example, myeloid cell-targeting agents may increase the number of responders to T-cell immune checkpoint inhibitors.^5^ As forms of myeloid cells, macrophages and dendritic cells (DC) are the focus of a number of developmental approaches^6–10^ and several agents, which impact these innate cells have been recently reviewed.^11^

PG545 (pixatimod, INN) is a small-molecule immunomodulatory agent with strong anti-heparanase activity^12–14^ that has been demonstrated to potently inhibit tumour-associated macrophages (TAM) in preclinical cancer models.^15,16^ Because heparanase plays a key role in the molecular decision-making that guides the cancer-promoting actions of TAM in pancreatic carcinoma,^17^ inhibition of this protein may be responsible for the inhibition of TAM in those studies. However, PG545 also exerts an immunostimulatory effect on DC, leading to the activation of natural killer (NK) cells capable of eradicating established lymphoma in mice.^18^ This effect was dependent on the presence of CD11c^+^ DC, IL-12 and TLR9^18^, but did not appear to be dependent on heparanase (unpublished data). Thus, PG545 modulates myeloid cells and represents a novel approach to enhance innate immunity against tumours.

As PG545 has shown significant activity in multiple tumour models, including pancreatic, colon, ovarian, breast and lung,^13,16,19–23^ the compound advanced into clinical testing for patients with advanced solid malignancies. Here, we report on the safety and activity of PG545 monotherapy in advanced cancer patients (NCT02042781).

## Materials and Methods

### Patients

Twenty-three adult patients with advanced, metastatic disease that had relapsed or was refractory to standard therapy or for which no effective standard therapy was available were enrolled across three sites in Australia. Additionally, patients had to have a performance status (Eastern Cooperative Oncology Group [ECOG]) ≤1 and adequate haematological, renal, cardiac and hepatic functions. All patients provided written informed consent. The study was approved by local human research ethics committees (HREC), the Royal Adelaide Hospital Research Ethics Committee (for RAH site) and Bellberry Limited (for the sites at Linear and Nucleus Network). The trial was conducted in accordance with the ICH Good Clinical Practice (GCP) guidelines and the Declaration of Helsinki.

### Study design and treatment

The study was an open-label, multicentre, phase 1 dose-escalation study using a 3 + 3 design. The primary objective was to determine the MTD of weekly administered PG545 via IV infusion. Secondary objectives were to characterize safety, tolerability, clinical activity, PK and PD biomarkers.

The initial cohort received 25 mg PG545 once weekly by 1-h IV infusion with dose escalation in subsequent cohorts following a pre-defined dose-escalation scheme: 50 mg, 100 mg and 150 mg. Treatment cycles were 28 days duration, with dosing occurring on days 1, 8, 15 and 22. Patients continued treatment until disease progression, unacceptable toxicity or withdrawal (either voluntarily or investigator-decision).

### Safety and efficacy assessments

All patients were assessed for safety, those who received at least one dose of PG545 were evaluated for efficacy. Safety assessment included the incidence of all adverse events (AEs), irrespective of relationship to study drug, according to the National Cancer Institute Common Terminology Criteria for Adverse Events, version 4.0, and the incidence of patients experiencing dose modifications and/or premature discontinuation of study drug. Dose-limiting toxicities (DLTs) used to determine the MTD were defined as occurring during Cycle 1 of treatment only and related to PG545 according to investigator assessment. If no DLT occurred in the first three subjects, the next three-subject cohort was enrolled at the next highest available dose level. If a single DLT occurred, an additional three subjects was enrolled at the same dose level. Dose escalation continued until two subjects of the cohort experienced a DLT; this was defined as the toxic dose level. The dose-escalation phase was completed and MTD determined by reducing the dose to the next lowest dose compared to the toxic dose and expanding this cohort to at least 6 subjects.

Subjects with measurable disease were assessed by computed tomography at baseline and every 8 weeks according to Response Evaluation Criteria in Solid Tumours (RECIST), version 1.1 criteria.

### Pharmacokinetics assessment

Plasma samples were obtained for the first (day 1) and fourth (day 22) doses, as follows: (1) pre-dosing (no more than 4 h prior to infusion start); (2) mid-point of infusion; prior to completion of infusion; 30 min post-infusion; then 2, 4, 6, 24, 48, 72, 144 and 168 h post-infusion. Samples were analysed for PG545 concentration using a validated liquid chromatography with tandem mass spectrometry (LC-MS/MS) assay.^13^ A population PK model was developed using NONMEM^®^ and individual non-compartmental analysis-type exposure parameter estimation was also performed.

### Pharmacodynamics assessment

Plasma and peripheral blood mononuclear cell (PBMC) samples were obtained at various points for PD assessment with the exception of the 25 mg cohort, where PBMC were not collected. Plasma samples were analysed for the concentration of various markers, including cytokines, chemokines and heparanase using commercial ELISA (LifeSpan Biosciences, Seattle, WA), and array kits (Quansys, Logan, UT).

Cryopreserved PBMC samples were analysed by flow cytometry for changes in abundance and activation status of DC and NK cells at day 3 post-treatment compared with pre-dose samples (day 1). Given the stimulatory nature of PG545 via TLR9 reported in mice,^18^ efforts were primarily focussed on the assessments of pDC and NK cells though certain subjects with available sample underwent additional analyses. To assess pDC, cells were stained with antibodies to HLADR-peCy7, CD303-FITC, IFNα-647 (BD Biosciences, Franklin Lakes, NJ). To assess NK cells, cells were stained with antibodies to CD335-PE, CD3-APC, IFNγ-FITC (BD Biosciences). All intracellular cytokine staining samples were stimulated by 100 ng/mL PMA, 100 ng/mL ionomycin and 5 μg/mL Brefeldin A for 4 h at 37 ˚C. Samples were acquired using FACSCanto flow cytometers (BD Biosciences).

## Results

### Patient demographics and baseline characteristics

Twenty-three patients were screened, enrolled, and received at least one dose of PG545 during the study. Baseline demographic and clinical characteristics of the population are summarised in Table 1.

**Table 1:** Patient demographics and baseline characteristics

Parameter	Overall	25 mg	50 mg	100 mg	150 mg
	*N* = 23	*N* = 3	*N* = 5	*N* = 6	*N* = 9
Median age, years (range)	62.0 (28, 76)	71.0 (38, 72)	68.0 (55, 76)	60.5 (28, 76)	60.0 (45, 75)
Male, %	61	33	40	67	78
Race, *n* (%)					
Caucasian	19 (83)	2 (67)	5 (100)	5 (83)	7 (78)
Asian	2 (9)	0	0	1 (17)	1 (11)
Other	2 (9)	1 (33)	0	0	1 (11)
ECOG, *n* (%)					
0	13 (57)	3 (100)	4 (80)	2 (33)	4 (44)
1	10 (43)	0	1 (20)	4 (67)	5 (56)
Stage of disease, *n* (%)					
IIIC	1 (4)	0	1 (20)	0	0
IV	19 (83)	2 (67)	4 (80)	5 (83)	8 (89)
Unknown	2 (9)	1 (33)	0	1 (17)	0
N/A	1 (4)	0	0	0	1 (11)
Prior therapy, *n* (%)					
Chemotherapy	18 (78)	0	4 (80)	6 (100)	8 (89)
Surgery	17 (74)	3 (100)	4 (80)	4 (67)	6 (67)
Radiotherapy	11 (48)	2 (67)	2 (40)	3 (50)	4 (44)
Other	13 (57)	2 (67)	2 (40)	5 (83)	4 (44)

### Safety

Dosing with PG545 began at 25 mg per patient per week (3 subjects) and escalated to 50 mg (5 subjects), 100 mg (6 subjects) and finally 150 mg (9 subjects). Details of enrollment and patient disposition are shown in the CONSORT diagram in the supplementary material (Supplementary Figure S1). Three DLT were observed in three patients within the 150 mg cohort during the first cycle and this was declared the toxic dose. The DLT were two cases of hypertension (3069 with no prior history and 3063 with history of hypertension but no anti-hypertensive medication at screening) and one case of epistaxis (with prior history). Although the hypertension occurred typically during or 1 h post-infusion and was transient, the events were typically associated with an increase in systolic pressure to 170 mm Hg and diastolic pressure to 100 mm Hg, with one subject experiencing a substantial increase in blood pressure (to 200/126 mm Hg). Both patients with hypertension continued on study after the DLT event, while the patient with epistaxis withdrew consent immediately following the DLT event. At that point, the 100 mg cohort was expanded and further three patients were treated. With no further DLT observed, the 100 mg dose was declared the MTD.

The most common treatment-related AE was hypertension with six occurrences, five in the 150 mg cohort (Table 2). All treatment-emergent AE are reported in supplementary material (Table S1). Of the 12 serious adverse events (SAEs), one was mild, nine were severe and two were fatal in grading (death due to disease progression). Six were assessed as possibly, likely, or certainly related to PG545 by the investigators (Table 3). Five of the SAEs resulted in patient withdrawal from the study or discontinuation of PG545 therapy. Only one patient (3069) experienced two different SAEs possibly related to PG545 (acute pulmonary oedema and severe hypertension).

**Table 2:** Treatment-related AEs (≥2 subjects)

Event Type	Overall	25 mg	50 mg	100 mg	150 mg
	*n* (%)	*n* (%)	*n* (%)	*n* (%)	*n* (%)
	*N* = 23	*N* = 3	*N* = 5	*N* = 6	*N* = 9
Hypertension	6 (26)	0	1 (20)	0	5 (56)
Chills	5 (22)	0	1 (20)	0	4 (44)
Fatigue	5 (22)	0	2 (40)	2 (33)	1 (11)
Pyrexia	5 (22)	0	3 (60)	1 (17)	1 (11)
Nausea	5 (22)	0	4 (80)	0	1 (11)
Decreased appetite	5 (22)	0	2 (40)	1 (17)	2 (22)
Infusion-related reaction	5 (22)	0	0	0	5 (56)
Diarrhoea	4 (17)	0	1 (20)	2 (33)	1 (11)
Hypercholesterolemia	2 (9)	0	0	2 (33)	0
Injection site reaction	2 (9)	2 (67)	0	0	0
Blood cholesterol increased	2 (9)	0	0	1 (17)	1 (11)
Headache	2 (9)	0	0	2 (33)	0

**Table 3:** Treatment-related severe AEs, AEs leading to discontinuation and serious AEs

Event Type	Overall	25 mg	50 mg	100 mg	150 mg
	*n* (%)	*n* (%)	*n* (%)	*n* (%)	*n* (%)
	*N* = 23	*N* = 3	*N* = 5	*N* = 6	*N* = 9
Related severe AEs					
Hypertension	5 (22)	0	0	0	5 (56)
Chills	2 (9)	0	1 (20)	0	1 (11)
Fatigue	2 (9)	0	1 (20)	1 (17)	0
Pyrexia	1 (4)	0	1 (20)	0	0
Blood cholesterol increased	1 (4)	0	0	0	1 (11)
Blood pressure increased	1 (4)	0	0	0	1 (11)
Acute pulmonary oedema	1 (4)	0	0	0	1 (11)
Epistaxis	1 (4)	0	0	0	1 (11)
Infusion-related reaction	1 (4)	0	0	0	1 (11)
Hyperlipidaemia	1 (4)	0	0	1 (17)	0
Related leading to discontinuation					
Pyrexia	1 (4)	0	1 (20)	0	0
Chills	1 (4)	0	1 (20)	0	0
Epistaxis	1 (4)	0	0	0	1 (11)
Acute pulmonary oedema	1 (4)	0	0	0	1 (11)
Hypertension	1 (4)	0	0	0	1 (11)
Related serious AE					
Acute pulmonary oedema	1 (4)	0	0	0	1 (11)
Epistaxis	1 (4)	0	0	0	1 (11)
Pyrexia	1 (4)	0	1 (20)	0	0
Infusion-related reaction	1 (4)	0	0	0	1 (11)
Hypovolaemia	1 (4)	0	0	1 (17)	0
Hypertension	1 (4)	0	0	0	1 (11)

### Clinical activity

**Fig. 1:**
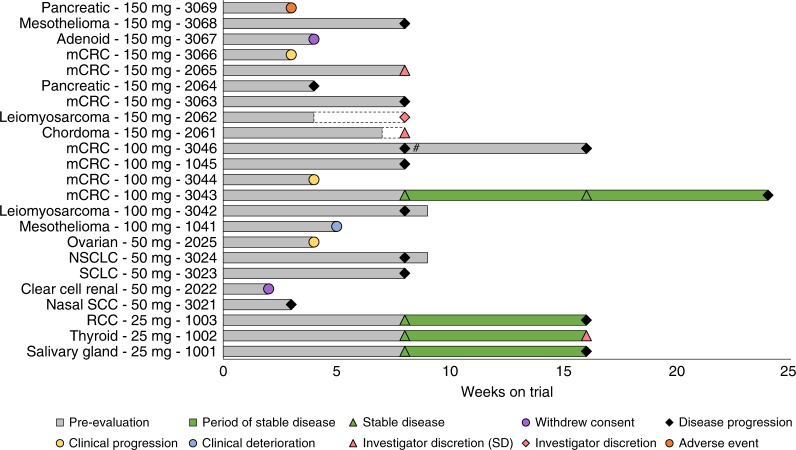
PG545 clinical activity. Cancer type, dose and subject ID indicated for the 23 subjects on trial and weeks on trial are shown in the swim plot. Dashed bars (subjects 2061 and 2062) indicate that subjects stopped receiving PG545 due to investigator discretion but stayed on study until 8 week assessment. ^#^ Subject stayed on study after PD erroneously classified as SD at 8 week. mCRC metastatic colorectal cancer, NSCLC non-small cell lung cancer, SCLC small cell lung cancer, SCC squamous cell carcinoma, RCC renal cell carcinoma

Sixteen patients had efficacy assessments during PG545 treatment and six of these had stable disease (SD) at 8 weeks as measured by RECIST 1.1 criteria. This ratio, six of sixteen assessed, represents a 38% disease control rate at 8 weeks (Fig. 1). The estimated median duration of SD for patients on the study was 57 days (95% confidence interval from 39 days to 62 days). Also included in the efficacy assessment were three patients with progressive disease (3021, 2062 and 2064) who did not receive the expected 8 doses scheduled. Seven patients were censored as part of the analysis, having no recorded findings of death or progressive disease.

### Pharmacokinetics

**Fig. 2:**
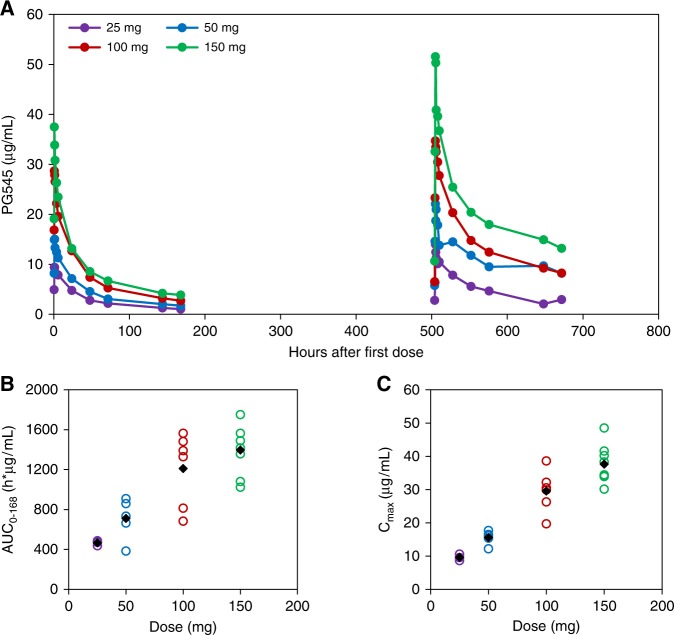
PG545 pharmacokinetics. Mean plasma PG545 concentration vs. time curves following first and fourth dose (**a**). Dose-proportionality plots for AUC_0–168_ (**b**) and *C*_max_ (**c**) yields a proportional exposure response to dosing, up to 100 mg. At the 150 mg dose level, exposure appears to be sub-proportional for AUC_0–168_ and *C*_max_ values. In **b**, **c**, the data for individual subjects are represented by open circles and cohort averages are represented as black diamonds

Cohort averaged data for PG545 plasma concentrations are plotted in Fig. 2a. A population pharmacokinetic model described the plasma concentrations after IV administration with good precision and little bias. PG545 had a small apparent central volume (Vc, 2.98 L), and apparent peripheral volume (Vp, 11.9 L) and a slow apparent clearance (CL, 0.0165 L/h), resulting in a long mean *t*_1/2_ estimated as 141 h across all subjects. Between subject variability was large for apparent peripheral volume (Vp, 61.2% CV) and CL (75.6% CV). The model and individual estimates for exposure (such as AUC and *C*_max_) indicated that the exposure was not dose proportional with individual exposure for the 150 mg-dosing group, which was being less than that predicted from lower-dosing groups if dose-proportionality was assumed. Individual estimates of area under the curve over the dosing interval (AUC_0–last_) after first dose indicated a general dose-proportionate exposure up to 100 mg (Fig. 2b and Table A1). Maximum observed PG545 concentration, derived from the measured concentration values (*C*_max_) was also broadly proportional up to 100 mg (Fig. 2c and Table S2).

### Pharmacodynamics

**Fig. 3:**
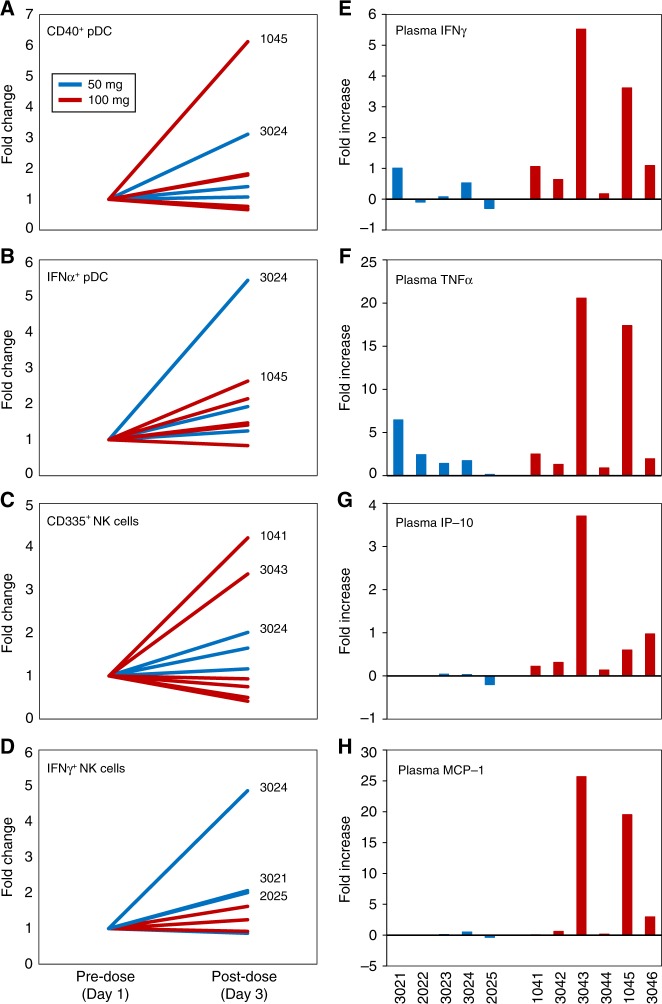
PG545 pharmacodynamics. Flow cytometry analysis of PBMC from the 50 and 100 mg cohorts showing increased expression of CD40 (**a**) and IFNα by pDC (**b**) and increased expression of NKp46 (**c**) and IFNγ by NK cells (**d**) after PG545 treatment. Patient numbers are indicated where increases are greater than two-fold. Analysis of plasma biomarkers shows increases in IFNγ (**e**), TNFα (**f**), IP-10 (**g**) and MCP-1 (**h**) after PG545 treatment in the 50 mg and 100 mg cohorts

Analysis of patient PBMC 2 days after the initial PG545 dose, revealed that, at the 50 and 100 mg dose levels, six of the ten subjects analysed in these cohorts exhibited at least two-fold increased numbers of either, or both, activated pDC or NK cells (Fig. 3). Two subjects had greater than two-fold increases in CD40^+^ pDC (3024 and 1045) with the same two and subject 1041 showing increases in IFNα expressing pDC (Fig. 3a,b). Three subjects, including two showing DC activation (3024 and 1041), and 3043 had increased numbers of the NKp46^+^ NK cells (Fig. 3c). There was also evidence of activation of NK cells, as indicated by increased IFNγ expression, in three subjects (Fig. 3d). In the 150 mg cohort, these parameters were unaffected or modestly responsive following treatment, though some subjects had increased expression of CD40 on conventional DCs (Supplementary Figure S2). PBMC were not collected for the 25 mg cohort.

Concentrations of selected signalling proteins were measured in subject plasma before and after the initial PG545 dose. Increases, of up to 25-fold, were found in plasma IFNγ, TNFα, IP-10 (CXCL10) and MCP-1 (CCL2) following PG545 treatment (Fig. 3e–h). Though cohort averages reveal PG545-induced elevations in IFNγ were similar between 100 mg and 150 mg cohorts, increases in TNFα, IL-6 and IL-8 responses were strikingly higher at 150 mg (Supplementary Figure S3). Changes in plasma concentrations for a range of cytokines, chemokines and potential pharmacodynamic markers are shown in the appendix (Supplementary Figures S3 and S4).

A summary of the immune modulation status following the first PG545 dose is provided in a matrix (Supplementary Table S3).

## Discussion

PG545 is a novel immunomodulatory agent, which has been demonstrated to possess potent anti-tumour activity in multiple preclinical models of cancer.^12–16,18–23^ Herein, we report on the first Phase 1 study of PG545 as an IV infusion in subjects with advanced cancer.

Treatment with PG545 was well tolerated, though paracetamol was required at doses above the 25 mg cohort to abrogate reactions associated with the infusion such as pyrexia and chills. Given that the immunostimulatory property of PG545 appears to be mediated via a TLR9-dependent mechanism,^18^ the flu-like symptoms and infusion reactions appear consistent with AEs associated with TLR9 agonists.^24^ However, the protective effect of paracetamol was lost once the dose reached 150 mg and additional pre-medications (cetirizine, amlodipine and hydrocortisone) had varying, but ultimately limited impact, on an emerging AE of acute transient hypertension during or shortly following infusion of PG545. Given the rapid onset and transient nature (<24 h) of the hypertensive episodes associated with PG545, it is unlikely that the effect is mediated through a similar mechanism as that described for anti-VEGF therapies.^25^ However, it is possible that activation of TLR9 could lead to hypertension.^26^ The elevation of plasma IL-8 levels in the 150 mg cohort could indicate that endothelial cells, which can express TLR9 and respond to TLR9 agonists, may be stimulated by this highest dose to release IL-8, a cytokine that can contribute to hypertension.^27,28^ Hypertension was not observed in the 100 mg cohort.

The lack of toxicity associated with the MTD was encouraging and the exposure achieved in these subjects (based on AUC_0–last_ values) was higher compared with mice receiving efficacious doses of PG545.^13,22^ Moreover, the AUC was sub-proportional for the 150 mg dose level. Simulation data using the model indicated that there were prolonged exposure and some accumulation after multiple weekly doses. Total exposure was reduced when simulation of a reduced dosing frequency after 4 weekly doses to once every 2 weeks was modelled. However, as the population mean minimum concentration over the dosing interval, based on this simulation, after 16 weeks (4 cycles) was approximately half (10 μg/mL versus 21 μg/mL) of that observed after once-weekly dosing at 100 mg (Supplementary Figure S5), any future modifications of dosing frequency could only be considered once exposure and its relationship to clinical activity are apparent.

The lack of objective responses using PG545 as a monotherapy in this clinical study is somewhat consistent with the published preclinical data in advanced disease settings, namely that only PG545 in combination with chemotherapy can eradicate large established tumours.^18^ Given the tremendous potential for combination approaches to extend the clinical success of immunotherapies^29^ and that maximal immunity is achieved only when both the innate and adaptive arms of the immune system work in concert,^30^ PG545 could also enhance clinical responses to T-cell-targeting immunotherapy such as immune checkpoint inhibitors by targeting innate immune cells. To that end, a new Phase 1b trial for metastatic pancreatic cancer is assessing the safety and tolerability of escalating doses of PG545 (25, 50 and 100 mg) with the anti-PD1 drug nivolumab (ANZCTR trial ID ACTRN12617001573347), which could further characterise the biologically optimal dose of PG545.

Preliminary pharmacodynamic analyses of PG545 identified changes in cell surface markers, cytokines and chemokines, which is consistent with preclinical findings^18^ and other innate stimulatory agents.^31–35^ At 50 and 100 mg dose levels, activation in pDCs of some patients was apparent either by elevations in CD40 expression or IFNα production, both of which are considered key targets for the treatment of cancer,^6,10,36,37^ while activation of NK cells was evident either by increased numbers of NKp46^+^ (CD335) cells or IFNγ production. Together with increases in several plasma cytokines and chemokines noted within 24 h of first PG545 treatment, for example, IFNγ, MCP-1 and IP-10 in the 100 mg cohort, such changes have been previously associated with immunomodulators such as TLR agonists.^31–35^ Though these changes may have some utility as biomarkers of inflammation and immune function, the new clinical study will examine not only T-cell-associated markers in PBMCs but also immune cell populations within the tumour microenvironment (TME), as this may be more predictive of clinical responses.

In conclusion, the study demonstrated that PG545 is well tolerated in patients with advanced solid malignancies and treatment was associated with changes in proposed pharmacodynamic markers. By modulating myeloid cells in the TME—as demonstrated in preclinical models via stimulation of DC to drive IL-12/TLR9/NK cell-dependent destruction of lymphoma in mice^18^ and inhibition of M2 macrophages and MDSCs^15,16^—PG545 offers a complementary approach for use in combination with existing therapies.

## Electronic supplementary material

Supplementary Material
