# Supplementary material for: A Phase I study of the novel immunomodulatory agent PG545 (pixatimod) in subjects with advanced solid tumours
Source: Br J Cancer. 2018 Mar 13;118(8):1035–41. doi: 10.1038/s41416-018-0006-0 (PMC5931096; doi:10.1038/s41416-018-0006-0)
Supplement: Supplementary file 1 — Supplementary Material [file 41416_2018_6_MOESM1_ESM.pdf]

Supplementary material

Inclusion and Exclusion Criteria

To be eligible for entry into the study, subjects were adults (≥ 18 years) with a life expectancy of at least 12 weeks, adequate organ function and measurable disease according to RECIST 1.1 and Eastern Cooperative Oncology Group (ECOG) performance status of 0 to 1.

Subjects with a known history of autoimmune conditions or previous exposure to immunotherapy agents were allowed into the study. One subject (001-041; 100 mg cohort) had a history of rheumatoid arthritis; subjects 003-023 (50 mg), 002-061 and 002-069 (150 mg) had a history of Type 2 diabetes; and subject 002-064 (150 mg) had a history of diabetes mellitus (subtype not identified). Subjects receiving prior immunotherapy were allowed onto the trial but there was no evidence of any subject receiving immunotherapy prior to enrolment.

Patients were ineligible for entry into the study if they had clinically significant non-malignant disease including, but not limited to, active clinically significant infection, myocardial infarction within 6 months prior to randomisation, cerebrovascular event or transient ischaemic attack within 12 months prior to randomisation or clinically significant gastrointestinal bleeding within 12 months prior to randomisation, previous clinically-significant bleeding from the tumour, uncontrolled diabetes or hypertension. Other exclusion criteria included major surgery within 6 weeks of randomisation, anti-cancer therapy within 4 weeks of Cycle 1 day 1 (excluding GnRH agonists for prostate cancer), palliative radiation for bone metastases within 2 weeks of Cycle 1 day 1, and patients with prior CNS metastases treated with only whole brain radiation therapy, patients with a history of allergy and/or hypersensitivity and/or other clinically significant adverse drug reaction to heparin or other anti-coagulant agents or a history of immune-mediated thrombocytopenia or other platelet abnormalities or other hereditary or acquired coagulopathies, or laboratory evidence of anti-heparin antibodies, or any previous history of having tested positive for anti-heparin antibodies.

Table S1: Treatment-Emergent Adverse Events by MedDRA System Organ Class and Preferred Term, Relationship to Study Treatment, and Severity (Safety Set)

Event Type	Mild		Moderate		Severe		Life threatening/Fatal	
	**Unrelated**	**Related**	**Unrelated**	**Related**	**Unrelated**	**Related**	**Unrelated**	**Related**
**At least one event**	**21 (91/3%)**	**16 (70.0%)**	**14 (60.9%)**	**13 (56.5%)**	**7 (30.4%)**	**11 (47.8%)**	**5 (21.7%)**	**0 (0.0%)**
**General disorders and administration site conditions**	**7 (30.4%)**	**11 (47.8%)**	**5 (21.7%)**	**4 (17.5%)**	**1 (4.3%)**	**4 (17.5%)**	**3 (13.0%)**	**0 (0.0%)**
Fatigue	2 (8.7%)	1 (4.3%)	3 (13.0%)	2 (8.7%)	0 (0.0%)	2 (8.7%)	1 (4.3%)	0 (0.0%)
Subject ID (dose (mg))	041 (100)<br>042 (100)	046 (100)	023 (50)<br>042 (100)<br>044 (100)	024 (50)<br>069 (150)		021 (50)<br>044 (100)	023 (50)	
Pyrexia	0 (0.0%)	5 (21.7%)	0 (0.0%)	1 (4.3%)	0 (0.0%)	1 (4.3%)	0 (0.0%)	0 (0.0%)
Subject ID (dose (mg))		022 (50)<br>024 (50)<br>046 (100)<br>063 (150)<br>069 (150)		021 (50)		002 (50)		
Chills	0 (0.0%)	2 (8.7%)	0 (0.0%)	2 (8.7%)	0 (0.0%)	2 (8.7%)	0 (0.0%)	0 (0.0%)
Subject ID (dose (mg))		065 (150)<br>068 (150)		022 (50)<br>069 (150)		002 (50)<br>063 (150)		
Chest pain	2 (8.7%)	0 (0.0%)	0 (0.0%)	0 (0.0%)	1 (4.3%)	0 (0.0%)	0 (0.0%)	0 (0.0%)
Subject ID (dose (mg))	001 (25)<br>024 (50)				024 (50)			

Event Type	Mild		Moderate		Severe		Life threatening/Fatal	
	Unrelated	Related	Unrelated	Related	Unrelated	Related	Unrelated	Related
Death	0 (0.0%)	0 (0.0%)	0 (0.0%)	0 (0.0%)	0 (0.0%)	0 (0.0%)	2 (8.7%)	0 (0.0%)
Subject ID (dose (mg))							044 (100)<br>066 (150)	
Injection site reaction	0 (0.0%)	2 (8.7%)	0 (0.0%)	0 (0.0%)	0 (0.0%)	0 (0.0%)	0 (0.0%)	0 (0.0%)
Subject ID (dose (mg))		001 (25)<br>002 (25)						
Malaise	1 (4.3%)	1 (4.3%)	1 (4.3%)	0 (0.0%)	0 (0.0%)	0 (0.0%)	0 (0.0%)	0 (0.0%)
Subject ID (dose (mg))	023 (50)	021 (50)	023 (50)					
Mucosal inflammation	2 (8.7%)	0 (0.0%)	0 (0.0%)	0 (0.0%)	0 (0.0%)	0 (0.0%)	0 (0.0%)	0 (0.0%)
Subject ID (dose (mg))	023 (50)<br>042 (100)							
Asthenia	0 (0.0%)	1 (4.3%)	0 (0.0%)	0 (0.0%)	0 (0.0%)	0 (0.0%)	0 (0.0%)	0 (0.0%)
Subject ID (dose (mg))		025 (50)						
Chest discomfort	1 (4.3%)	0 (0.0%)	0 (0.0%)	0 (0.0%)	0 (0.0%)	0 (0.0%)	0 (0.0%)	0 (0.0%)
Subject ID (dose (mg))	045 (100)							
Gait disturbance	0 (0.0%)	0 (0.0%)	1 (4.3%)	0 (0.0%)	0 (0.0%)	0 (0.0%)	0 (0.0%)	0 (0.0%)
Subject ID (dose (mg))			044 (100)					
Influenza like illness	1 (4.3%)	0 (0.0%)	0 (0.0%)	0 (0.0%)	0 (0.0%)	0 (0.0%)	0 (0.0%)	0 (0.0%)
Subject ID (dose (mg))	002 (25)							
Pain	1 (4.3%)	0 (0.0%)	0 (0.0%)	0 (0.0%)	0 (0.0%)	0 (0.0%)	0 (0.0%)	0 (0.0%)
Subject ID (dose (mg))	023 (50)							

Event Type	Mild		Moderate		Severe		Life threatening/Fatal	
	**Unrelated**	**Related**	**Unrelated**	**Related**	**Unrelated**	**Related**	**Unrelated**	**Related**
**Gastrointestinal disorders**	**13 (56.5%)**	**8 (34.8%)**	**6 (26.1%)**	**4 (17.4%)**	**2 (8.7%)**	**0 (0.0%)**	**0 (0.0%)**	**0 (0.0%)**
Nausea	3 (13.0%)	4 (17.5%)	2 (8.7%)	1 (4.3%)	0 (0.0%)	0 (0.0%)	0 (0.0%)	0 (0.0%)
Subject ID (dose (mg))	024 (50)<br>066 (150)<br>069 (150)	022 (50)<br>023 (50)<br>024 (50)<br>063 (150)	023 (50)<br>044 (100)	021 (50)				
Constipation	5 (21.7%)	1 (4.3%)	1 (4.3%)	1 (4.3%)	1 (4.3%)	0 (0.0%)	0 (0.0%)	0 (0.0%)
Subject ID (dose (mg))	021 (50)<br>023 (50)<br>024 (50)<br>042 (100)<br>062 (150)	069 (150)	042 (100)	063 (150)	021 (50)			
Diarrhoea	3 (13.0%)	4 (17.5%)	0 (0.0%)	1 (4.3%)	0 (0.0%)	0 (0.0%)	0 (0.0%)	0 (0.0%)
Subject ID (dose (mg))	023 (50)<br>045 (100)<br>069 (150)	024 (50)<br>044 (100)<br>046 (100)<br>068 (150)		044 (100)				
Abdominal pain	2 (8.7%)	0 (0.0)%	3 (13.0%)	0 (0.0%)	0 (0.0%)	0 (0.0%)	0 (0.0%)	0 (0.0%)
Subject ID (dose (mg))	025 (50)<br>063 (150)		042 (100)<br>062 (150)<br>065 (150)					

Event Type	Mild		Moderate		Severe		Life threatening/Fatal	
	Unrelated	Related	Unrelated	Related	Unrelated	Related	Unrelated	Related
Vomiting	5 (21.7%)	0 (0.0%)	0 (0.0%)	1 (4.3%)	0 (0.0%)	0 (0.0%)	0 (0.0%)	0 (0.0%)
Subject ID (dose (mg))	025 (50)<br>041 (100)<br>045 (100)<br>066 (150)<br>068 (150)			021 (50)				
Abdominal distension	1 (4.3%)	1 (4.3%)	1 (4.3%)	0 (0.0%)	0 (0.0%)	0 (0.0%)	0 (0.0%)	0 (0.0%)
Subject ID (dose (mg))	025 (50)	023 (50)	025 (50)					
Abdominal pain lower	0 (0.0%)	1 (4.3%)	1 (4.3%)	0 (0.0%)	0 (0.0%)	0 (0.0%)	0 (0.0%)	0 (0.0%)
Subject ID (dose (mg))		022 (50)	042 (100)					
Abdominal pain upper	1 (4.3%)	0 (0.0%)	2 (8.7%)	0 (0.0%)	0 (0.0%)	0 (0.0%)	0 (0.0%)	0 (0.0%)
Subject ID (dose (mg))	023 (50)		023 (50)<br>044 (100)					
Dyspepsia	1 (4.3%)	1 (4.3%)	0 (0.0%)	0 (0.0%)	0 (0.0%)	0 (0.0%)	0 (0.0%)	0 (0.0%)
Subject ID (dose (mg))	066 (150)	024 (50)						
Ascites	1 (4.3%)	0 (0.0%)	0 (0.0%)	0 (0.0%)	0 (0.0%)	0 (0.0%)	0 (0.0%)	0 (0.0%)
Subject ID (dose (mg))	062 (150)							
Dry mouth	1 (4.3%)	0 (0.0%)	1 (4.3%)	0 (0.0%)	0 (0.0%)	0 (0.0%)	0 (0.0%)	0 (0.0%)
Subject ID (dose (mg))	044 (100)		044 (100)					
Gastroesophageal reflux disease	1 (4.3%)	0 (0.0%)	0 (0.0%)	0 (0.0%)	0 (0.0%)	0 (0.0%)	0 (0.0%)	0 (0.0%)
Subject ID (dose (mg))	045 (100)							

Event Type	Mild		Moderate		Severe		Life threatening/Fatal	
	**Unrelated**	**Related**	**Unrelated**	**Related**	**Unrelated**	**Related**	**Unrelated**	**Related**
Haematochezia	1 (4.3%)	0 (0.0%)	0 (0.0%)	0 (0.0%)	0 (0.0%)	0 (0.0%)	0 (0.0%)	0 (0.0%)
Subject ID (dose (mg))	045 (100)							
Intestinal obstruction	0 (0.0%)	0 (0.0%)	1 (4.3%)	0 (0.0%)	0 (0.0%)	0 (0.0%)	0 (0.0%)	0 (0.0%)
Subject ID (dose (mg))			025 (50)					
Small intestinal obstruction	0 (0.0%)	0 (0.0%)	0 (0.0%)	0 (0.0%)	1 (4.3%)	0 (0.0%)	0 (0.0%)	0 (0.0%)
Subject ID (dose (mg))					025 (50)			
**Metabolism and nutritional disorders**	**2 (8.7%)**	**5 (21.7%)**	**4 (17.4%)**	**4 (17.4%)**	**2 (8.7%)**	**1 (4.3%)**	**1 (4.3%)**	**0 (0.0%)**
Decreased appetite	1 (4.3%)	4 (17.4%)	3 (13.0%)	2 (8.7%)	0 (0.0%)	0 (0.0%)	0 (0.0%)	0 (0.0%)
Subject ID (dose (mg))	023 (50)	022 (50)<br>046 (100)<br>068 (150)<br>069 (150)	023 (50)<br>025 (50)<br>044 (100)	021 (50)<br>024 (50)				
Dehydration	0 (0.0%)	0 (0.0%)	0 (0.0%)	1 (4.3%)	1 (4.3%)	0 (0.0%)	0 (0.0%)	0 (0.0%)
Subject ID (dose (mg))				021 (50)	023 (50)			
Hypercholesterolaemia	0 (0.0%)	1 (4.3%)	0 (0.0%)	2 (8.7%)	0 (0.0%)	0 (0.0%)	0 (0.0%)	0 (0.0%)
Subject ID (dose (mg))		045 (100)		042 (100)<br>045 (100)				
Hypercalcaemia	1 (4.3%)	0 (0.0%)	0 (0.0%)	0 (0.0%)	0 (0.0%)	0 (0.0%)	0 (0.0%)	0 (0.0%)
Subject ID (dose (mg))	069 (150)							

Event Type	Mild		Moderate		Severe		Life threatening/Fatal	
	Unrelated	Related	Unrelated	Related	Unrelated	Related	Unrelated	Related
Hyperlipidaemia	0 (0.0%)	0 (0.0%)	0 (0.0%)	1 (4.3%)	0 (0.0%)	1 (4.3%)	0 (0.0%)	0 (0.0%)
Subject ID (dose (mg))				042 (100)		042 (100)		
Hyperuricaemia	0 (0.0%)	0 (0.0%)	0 (0.0%)	0 (0.0%)	0 (0.0%)	0 (0.0%)	1 (4.3%)	0 (0.0%)
Subject ID (dose (mg))							044 (100)	
Hypoalbuminaemia	0 (0.0%)	0 (0.0%)	1 (4.3%)	0 (0.0%)	0 (0.0%)	0 (0.0%)	0 (0.0%)	0 (0.0%)
Subject ID (dose (mg))			062 (150)					
Hypokalaemia	1 (4.3%)	0 (0.0%)	0 (0.0%)	0 (0.0%)	0 (0.0%)	0 (0.0%)	0 (0.0%)	0 (0.0%)
Subject ID (dose (mg))	069 (150)							
Hyponatraemia	0 (0.0%)	0 (0.0%)	0 (0.0%)	0 (0.0%)	1 (4.3%)	0 (0.0%)	0 (0.0%)	0 (0.0%)
Subject ID (dose (mg))					044 (100)			
Hypovolaemia	0 (0.0%)	1 (4.3%)	0 (0.0%)	0 (0.0%)	0 (0.0%)	0 (0.0%)	0 (0.0%)	0 (0.0%)
Subject ID (dose (mg))		046 (100)						
**Musculoskeletal and connective tissue disorders**	**5 (21.7%)**	**2 (8.7%)**	**6 (26.1%)**	**0 (0.0%)**	**0 (0.0%)**	**0 (0.0%)**	**0 (0.0%)**	**0 (0.0%)**
Back pain	2 (8.7%)	1 (4.3%)	3 (13.0%)	0 (0.0%)	0 (0.0%)	0 (0.0%)	0 (0.0%)	0 (0.0%)
Subject ID (dose (mg))	043 (100)<br>065 (150)	024 (50)	042 (100)<br>062 (150)<br>063 (150)					
Musculoskeletal pain	0 (0.0%)	1 (4.3%)	1 (4.3%)	0 (0.0%)	0 (0.0%)	0 (0.0%)	0 (0.0%)	0 (0.0%)
Subject ID (dose (mg))		022 (50)	068 (150)					

Event Type	Mild		Moderate		Severe		Life threatening/Fatal	
	Unrelated	Related	Unrelated	Related	Unrelated	Related	Unrelated	Related
Pain in extremity	2 (8.7%)	0 (0.0%)	1 (4.3%)	0 (0.0%)	0 (0.0%)	0 (0.0%)	0 (0.0%)	0 (0.0%)
Subject ID (dose (mg))	042 (100)<br>046 (100)		042 (100)					
Arthralgia	0 (0.0%)	0 (0.0%)	1 (4.3%)	0 (0.0%)	0 (0.0%)	0 (0.0%)	0 (0.0%)	0 (0.0%)
Subject ID (dose (mg))			024 (50)					
Groin pain	0 (0.0%)	0 (0.0%)	1 (4.3%)	0 (0.0%)	0 (0.0%)	0 (0.0%)	0 (0.0%)	0 (0.0%)
Subject ID (dose (mg))			065 (150)					
Musculoskeletal chest pain	1 (4.3%)	0 (0.0%)	0 (0.0%)	0 (0.0%)	0 (0.0%)	0 (0.0%)	0 (0.0%)	0 (0.0%)
Subject ID (dose (mg))	044 (100)							
**Nervous system disorders**	**5 (21.7%)**	**3 (13.0%)**	**2 (8.7%)**	**1 (4.3%)**	**0 (0.0%)**	**0 (0.0%)**	**0 (0.0%)**	**0 (0.0%)**
Headache	1 (4.3%)	2 (8.7%)	2 (8.7%)	0 (0.0%)	0 (0.0%)	0 (0.0%)	0 (0.0%)	0 (0.0%)
Subject ID (dose (mg))	045 (100)	044 (100)<br>046 (100)	044 (100)<br>061 (150)					
Lethargy	2 (8.7%)	0 (0.0%)	0 (0.0%)	1 (4.3%)	0 (0.0%)	0 (0.0%)	0 (0.0%)	0 (0.0%)
Subject ID (dose (mg))	001 (25)<br>023 (50)			021 (50)				
Paraesthesia	1 (4.3%)	0 (0.0%)	0 (0.0%)	0 (0.0%)	0 (0.0%)	0 (0.0%)	0 (0.0%)	0 (0.0%)
Subject ID (dose (mg))	041 (100)<br>043 (100)							
Dizziness	0 (0.0%)	1 (4.3%)	0 (0.0%)	0 (0.0%)	0 (0.0%)	0 (0.0%)	0 (0.0%)	0 (0.0%)
Subject ID (dose (mg))		046 (100)						

Event Type	Mild		Moderate		Severe		Life threatening/Fatal	
	**Unrelated**	**Related**	**Unrelated**	**Related**	**Unrelated**	**Related**	**Unrelated**	**Related**
Dysgeusia	0 (0.0%)	1 (4.3%)	0 (0.0%)	0 (0.0%)	0 (0.0%)	0 (0.0%)	0 (0.0%)	0 (0.0%)
Subject ID (dose (mg))		021 (50)						
**Respiratory, thoracic and mediastinal disorders**	**6 (26.1%)**	**1 (4.3%)**	**4 (17.4%)**	**1 (4.3%)**	**0 (0.0%)**	**2 (8.7%)**	**0 (0.0%)**	**0 (0.0%)**
Dyspnoea	5 (21.7%)	0 (0.0%)	3 (13.0%)	1 (4.3%)	0 (0.0%)	0 (0.0%)	0 (0.0%)	0 (0.0%)
Subject ID (dose (mg))	043 (100)<br>044 (100)<br>063 (150)<br>068 (150)<br>069 (150)		023 (50)<br>044 (100)<br>068 (150)	066 (150)				
Cough	2 (8.7%)	1 (4.3%)	1 (4.3%)	0 (0.0%)	0 (0.0%)	0 (0.0%)	0 (0.0%)	0 (0.0%)
Subject ID (dose (mg))	044 (100)<br>063 (150)	068 (150)	066 (150)					
Acute pulmonary oedema	0 (0.0%)	0 (0.0%)	0 (0.0%)	0 (0.0%)	0 (0.0%)	1 (4.3%)	0 (0.0%)	0 (0.0%)
Subject ID (dose (mg))						069 (150)		
Epistaxis	0 (0.0%)	0 (0.0%)	0 (0.0%)	0 (0.0%)	0 (0.0%)	1 (4.3%)	0 (0.0%)	0 (0.0%)
Subject ID (dose (mg))						067 (150)		
Oropharyngeal pain	1 (4.3%)	0 (0.0%)	0 (0.0%)	0 (0.0%)	0 (0.0%)	0 (0.0%)	0 (0.0%)	0 (0.0%)
Subject ID (dose (mg))	002 (25)							

Event Type	Mild		Moderate		Severe		Life threatening/Fatal	
	**Unrelated**	**Related**	**Unrelated**	**Related**	**Unrelated**	**Related**	**Unrelated**	**Related**
**Investigations**	**3 (13.0%)**	**3 (13.0%)**	**3 (13.0%)**	**1 (4.3%)**	**2 (8.7%)**	**2 (8.7%)**	**3 (13.0%)**	**0 (0.0%)**
GGT increased	0 (0.0%)	0 (0.0%)	1 (4.3%)	1 (4.3%)	1 (4.3%)	0 (0.0%)	3 (13.0%)	0 (0.0%)
Subject ID (dose (mg))			069 (150)	068 (150)	044 (100)		044 (100)<br>062 (150)<br>064 (150)	
Blood ALP increased	1 (4.3%)	1 (4.3%)	1 (4.3%)	0 (0.0%)	2 (8.7%)	0 (0.0%)	0 (0.0%)	0 (0.0%)
Subject ID (dose (mg))	069 (150)	068 (150)	046 (100)		044 (100)<br>046 (100)			
Blood cholesterol increased	0 (0.0%)	1 (4.3%)	0 (0.0%)	0 (0.0%)	0 (0.0%)	1 (4.3%)	0 (0.0%)	0 (0.0%)
Subject ID (dose (mg))		046 (100)				065 (150)		
Neutrophil count increased	1 (4.3%)	0 (0.0%)	1 (4.3%)	0 (0.0%)	0 (0.0%)	0 (0.0%)	0 (0.0%)	0 (0.0%)
Subject ID (dose (mg))	044 (100)		069 (150)					
White blood cell count increased	1 (4.3%)	0 (0.0%)	1 (4.3%)	0 (0.0%)	0 (0.0%)	0 (0.0%)	0 (0.0%)	0 (0.0%)
Subject ID (dose (mg))	044 (100)		069 (150)					
aPTT prolonged	1 (4.3%)	0 (0.0%)	0 (0.0%)	0 (0.0%)	0 (0.0%)	0 (0.0%)	0 (0.0%)	0 (0.0%)
Subject ID (dose (mg))	046 (100)							
AST increased	0 (0.0%)	0 (0.0%)	1 (4.3%)	0 (0.0%)	1 (4.3%)	0 (0.0%)	0 (0.0%)	0 (0.0%)
Subject ID (dose (mg))			044 (100)		044 (100)			
Blood bilirubin increased	0 (0.0%)	0 (0.0%)	1 (4.3%)	0 (0.0%)	0 (0.0%)	0 (0.0%)	0 (0.0%)	0 (0.0%)
Subject ID (dose (mg))			044 (100)					

Event Type	Mild		Moderate		Severe		Life threatening/Fatal	
	**Unrelated**	**Related**	**Unrelated**	**Related**	**Unrelated**	**Related**	**Unrelated**	**Related**
Blood creatinine increased	1 (4.3%)	0 (0.0%)	0 (0.0%)	0 (0.0%)	0 (0.0%)	0 (0.0%)	0 (0.0%)	0 (0.0%)
Subject ID (dose (mg))	044 (100)							
Blood LDH increased	0 (0.0%)	0 (0.0%)	0 (0.0%)	0 (0.0%)	0 (0.0%)	0 (0.0%)	0 (0.0%)	0 (0.0%)
Subject ID (dose (mg))								
Blood pressure increased	0 (0.0%)	0 (0.0%)	0 (0.0%)	0 (0.0%)	0 (0.0%)	1 (4.3%)	0 (0.0%)	0 (0.0%)
Subject ID (dose (mg))						063 (150)		
Blood triglycerides increased	0 (0.0%)	1 (4.3%)	0 (0.0%)	0 (0.0%)	0 (0.0%)	0 (0.0%)	0 (0.0%)	0 (0.0%)
Subject ID (dose (mg))		046 (100)						
Low density lipoprotein increased	0 (0.0%)	1 (4.3%)	0 (0.0%)	0 (0.0%)	0 (0.0%)	0 (0.0%)	0 (0.0%)	0 (0.0%)
Subject ID (dose (mg))		046 (100)						
Oxygen saturation decreased	0 (0.0%)	1 (4.3%)	0 (0.0%)	0 (0.0%)	0 (0.0%)	0 (0.0%)	0 (0.0%)	0 (0.0%)
Subject ID (dose (mg))		063 (150)						
Platelet count increased	0 (0.0%)	0 (0.0%)	1 (4.3%)	0 (0.0%)	0 (0.0%)	0 (0.0%)	0 (0.0%)	0 (0.0%)
Subject ID (dose (mg))			069 (150)					
**Psychiatric disorders**	**5 (21.7%)**	**0 (0.0%)**	**2 (8.7%)**	**0 (0.0%)**	**0 (0.0%)**	**0 (0.0%)**	**0 (0.0%)**	**0 (0.0%)**
Depression	3 (13.0%)	0 (0.0%)	1 (4.3%)	0 (0.0%)	0 (0.0%)	0 (0.0%)	0 (0.0%)	0 (0.0%)
Subject ID (dose (mg))	023 (50)<br>042 (100)<br>043 (100)		024 (50)					

Event Type	Mild		Moderate		Severe		Life threatening/Fatal	
	**Unrelated**	**Related**	**Unrelated**	**Related**	**Unrelated**	**Related**	**Unrelated**	**Related**
Insomnia	3 (13.0%)	0 (0.0%)	1 (4.3%)	0 (0.0%)	0 (0.0%)	0 (0.0%)	0 (0.0%)	0 (0.0%)
Subject ID (dose (mg))	024 (50)<br>043 (100)<br>068 (150)		044 (100)					
Anxiety	2 (8.7%)	0 (0.0%)	0 (0.0%)	0 (0.0%)	0 (0.0%)	0 (0.0%)	0 (0.0%)	0 (0.0%)
Subject ID (dose (mg))	024 (50)<br>045 (100)							
**Vascular disorders**	**2 (8.7%)**	**1 (4.3%)**	**0 (0.0%)**	**2 (8.7%)**	**0 (0.0%)**	**5 (21.7%)**	**0 (0.0%)**	**0 (0.0%)**
Hypertension	0 (0.0%)	0 (0.0%)	0 (0.0%)	2 (8.7%)	0 (0.0%)	5 (21.7%)	0 (0.0%)	0 (0.0%)
Subject ID (dose (mg))				021 (50)<br>061 (150)		061 (150)<br>062 (150)<br>064 (150)<br>065 (150)<br>069 (150)		
Deep vein thrombosis	1 (4.3%)	0 (0.0%)	0 (0.0%)	0 (0.0%)	0 (0.0%)	0 (0.0%)	0 (0.0%)	0 (0.0%)
Subject ID (dose (mg))	022 (50)							
Hypertension	1 (4.3%)	0 (0.0%)	0 (0.0%)	0 (0.0%)	0 (0.0%)	0 (0.0%)	0 (0.0%)	0 (0.0%)
Subject ID (dose (mg))	021 (50)							
Peripheral coldness	0 (0.0%)	1 (4.3%)	0 (0.0%)	0 (0.0%)	0 (0.0%)	0 (0.0%)	0 (0.0%)	0 (0.0%)
Subject ID (dose (mg))		069 (150)						

Event Type	Mild		Moderate		Severe		Life threatening/Fatal	
	**Unrelated**	**Related**	**Unrelated**	**Related**	**Unrelated**	**Related**	**Unrelated**	**Related**
**Injury, poisoning and procedural complications**	**0 (0.0%)**	**2 (8.7%)**	**0 (0.0%)**	**5 (21.7%)**	**0 (0.0%)**	**1 (4.3%)**	**0 (0.0%)**	**0 (0.0%)**
Infusion related reaction	0 (0.0%)	1 (4.3%)	0 (0.0%)	4 (17.4%)	0 (0.0%)	1 (4.3%)	0 (0.0%)	0 (0.0%)
Subject ID (dose (mg))		068 (150)		061 (150)<br>062 (150)<br>064 (150)<br>068 (150)		063 (150)		
Fall	0 (0.0%)	1 (4.3%)	0 (0.0%)	1 (4.3%)	0 (0.0%)	0 (0.0%)	0 (0.0%)	0 (0.0%)
Subject ID (dose (mg))		021 (50)		021 (50)				
**Infections and infestations**	**4 (17.4%)**	**0 (0.0%)**	**1 (4.3%)**	**0 (0.0%)**	**1 (4.3%)**	**0 (0.0%)**	**0 (0.0%)**	**0 (0.0%)**
Upper respiratory tract infection	2 (8.7%)	0 (0.0%)	1 (4.3%)	0 (0.0%)	0 (0.0%)	0 (0.0%)	0 (0.0%)	0 (0.0%)
Subject ID (dose (mg))	003 (25)<br>067 (150)		067 (150)					
Fungal infection	1 (4.3%)	0 (0.0%)	0 (0.0%)	0 (0.0%)	0 (0.0%)	0 (0.0%)	0 (0.0%)	0 (0.0%)
Subject ID (dose (mg))	002 (25)							
Lower respiratory tract infection	0 (0.0%)	0 (0.0%)	0 (0.0%)	0 (0.0%)	1 (4.3%)	0 (0.0%)	0 (0.0%)	0 (0.0%)
Subject ID (dose (mg))					069 (150)			
Vulvovaginal candidiasis	1 (4.3%)	0 (0.0%)	0 (0.0%)	0 (0.0%)	0 (0.0%)	0 (0.0%)	0 (0.0%)	0 (0.0%)
Subject ID (dose (mg))	024 (50)							

Event Type	Mild		Moderate		Severe		Life threatening/Fatal	
	**Unrelated**	**Related**	**Unrelated**	**Related**	**Unrelated**	**Related**	**Unrelated**	**Related**
**Skin and subcutaneous tissue disorders**	**3 (13.0%)**	**2 (8.7%)**	**0 (0.0%)**	**0 (0.0%)**	**0 (0.0%)**	**0 (0.0%)**	**0 (0.0%)**	**0 (0.0%)**
Dry skin	1 (4.3%)	0 (0.0%)	0 (0.0%)	0 (0.0%)	0 (0.0%)	0 (0.0%)	0 (0.0%)	0 (0.0%)
Subject ID (dose (mg))	046 (100)							
Onycholysis	1 (4.3%)	0 (0.0%)	0 (0.0%)	0 (0.0%)	0 (0.0%)	0 (0.0%)	0 (0.0%)	0 (0.0%)
Subject ID (dose (mg))	024 (50)							
Pruritus	1 (4.3%)	0 (0.0%)	0 (0.0%)	0 (0.0%)	0 (0.0%)	0 (0.0%)	0 (0.0%)	0 (0.0%)
Subject ID (dose (mg))	045 (100)							
Rash	0 (0.0%)	1 (4.3%)	0 (0.0%)	0 (0.0%)	0 (0.0%)	0 (0.0%)	0 (0.0%)	0 (0.0%)
Subject ID (dose (mg))		002 (25)						
Rash erythematous	0 (0.0%)	1 (4.3%)	0 (0.0%)	0 (0.0%)	0 (0.0%)	0 (0.0%)	0 (0.0%)	0 (0.0%)
Subject ID (dose (mg))		067 (150)						
**Blood and lymphatic system disorders**	**0 (0.0%)**	**1 (4.3%)**	**2 (8.7%)**	**0 (0.0%)**	**1 (4.3%)**	**0 (0.0%)**	**0 (0.0%)**	**0 (0.0%)**
Anaemia	0 (0.0%)	1 (4.3%)	2 (8.7%)	0 (0.0%)	1 (4.3%)	0 (0.0%)	0 (0.0%)	0 (0.0%)
Subject ID (dose (mg))		045 (100)	044 (100)<br>069 (150)		069 (150)			
**Eye disorders**	**0 (0.0%)**	**1 (4.3%)**	**1 (4.3%)**	**0 (0.0%)**	**0 (0.0%)**	**0 (0.0%)**	**0 (0.0%)**	**0 (0.0%)**
Diplopia	0 (0.0%)	1 (4.3%)	1 (4.3%)	0 (0.0%)	0 (0.0%)	0 (0.0%)	0 (0.0%)	0 (0.0%)
Subject ID (dose (mg))		041 (100)	061 (150)					

Event Type	Mild		Moderate		Severe		Life threatening/Fatal	
	**Unrelated**	**Related**	**Unrelated**	**Related**	**Unrelated**	**Related**	**Unrelated**	**Related**
**Renal and urinary disorders**	**0 (0.0%)**	**0 (0.0%)**	**0 (0.0%)**	**0 (0.0%)**	**1 (4.3%)**	**0 (0.0%)**	**0 (0.0%)**	**0 (0.0%)**
Dysuria	0 (0.0%)	0 (0.0%)	0 (0.0%)	0 (0.0%)	1 (4.3%)	0 (0.0%)	0 (0.0%)	0 (0.0%)
Subject ID (dose (mg))					021 (50)			

Table S2. Individual Non-compartmental Analysis (NCA) type Exposure Parameter Estimation. Mean of individual subject NCA type exposure parameter values by dose group are presented below.

**Dose amount**	**Dose number**	**Cmax (g/mL)**	**$t_{1/2}$ (h)**	**$AUC_{0\text{-}last}$ (h*µg/mL)**	**$AUC_{0\text{-}96}$ (h*µg/mL)**	**AUC_{tau} (h*µg/mL)**
25 mg	1	9.60	148.99	465.17	339.09	432.04
	4	12.44	122.81	610.54	588.96	840.89
50 mg	1	15.58	182.89	709.57	539.25	695.36
	4	21.73	228.50	1688.54	1116.93	1638.67
100 mg	1	29.53	133.15	1209.99	973.99	1212.72
	4	35.55	133.15	2381.44	1605.65	2248.33
150 mg	1	37.06	116.93	1441.18	1189.19	1496.42
	4	48.63	127.54	2781.3	2226.06	3191.28

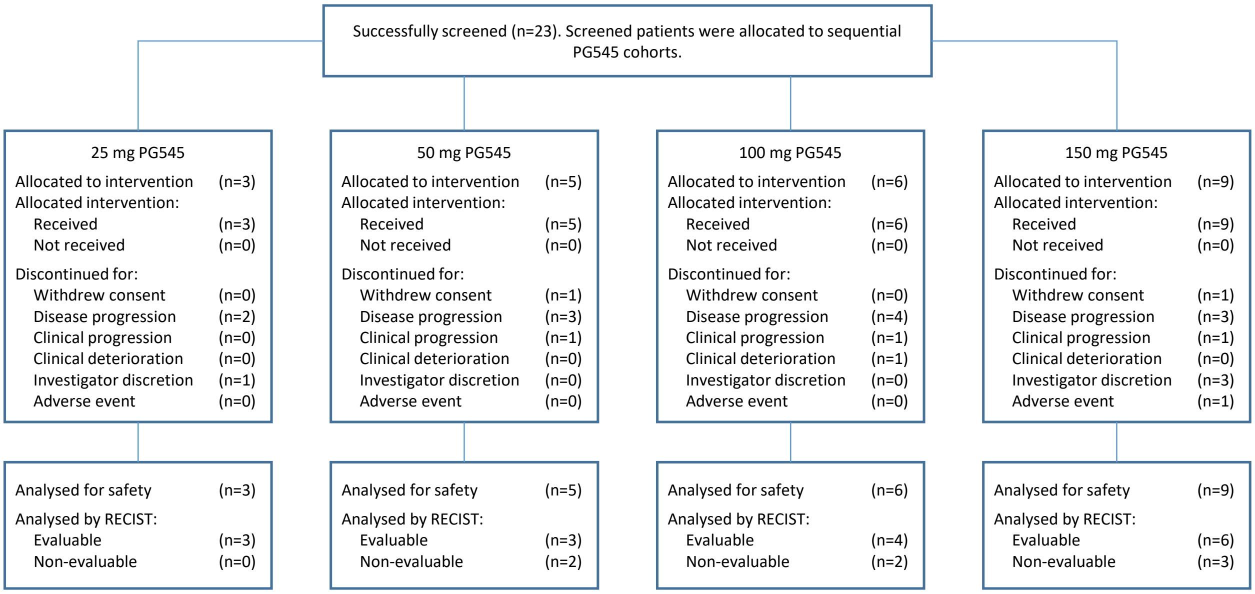

Figure S1. Disposition of subjects in study PG545102

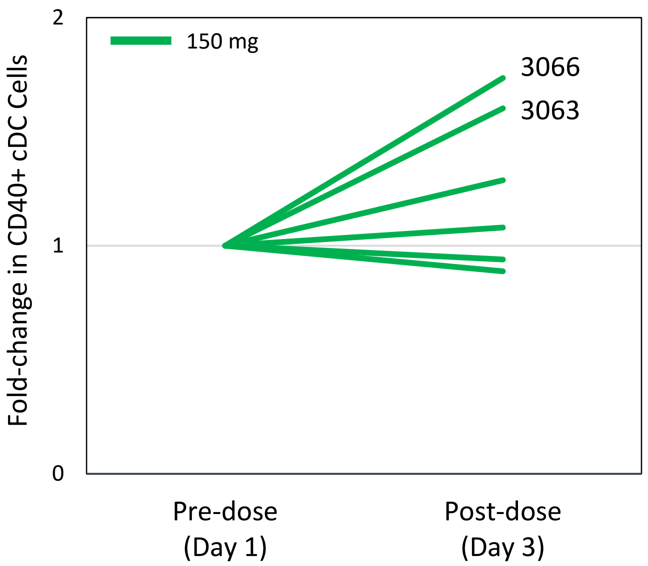

Figure S2. Flow cytometry analysis of PBMC from subjects of the 150 mg cohort showing increased expression of CD40 by cDC (CD11c+, HLA-DR+) in some subjects. To assess cDC, cells were stained with antibodies to HLADR-peCy7, CD11c-peCF594, CD40-APC (BD Biosciences, Franklin Lakes, NJ). Samples were acquired using a 5-laser BD LSR Fortessa X-20 flow cytometer (BD Biosciences).

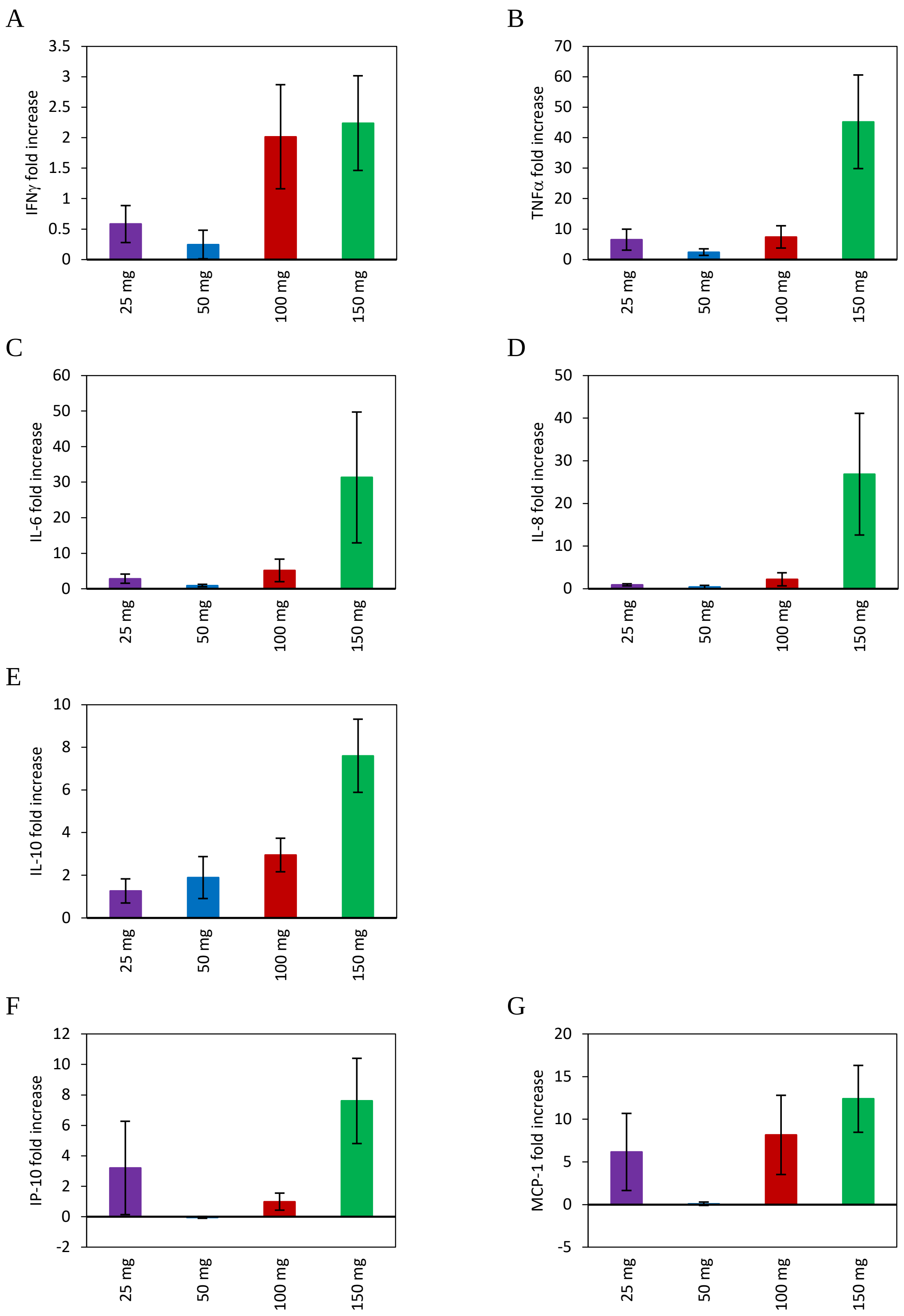

Figure S3. Modulation of various cytokines in patient plasma during PG545 treatment. Fold increases during the 24 h following the first PG545 dose are plotted for IFNγ (A), TNFα (B), IL-6 (C), IL-8 (D), IL-10 (E), IP-10 (F) and MCP-1 (G). Averages include all subjects analysed for each cohort for that particular analyte and error bars are standard errors.

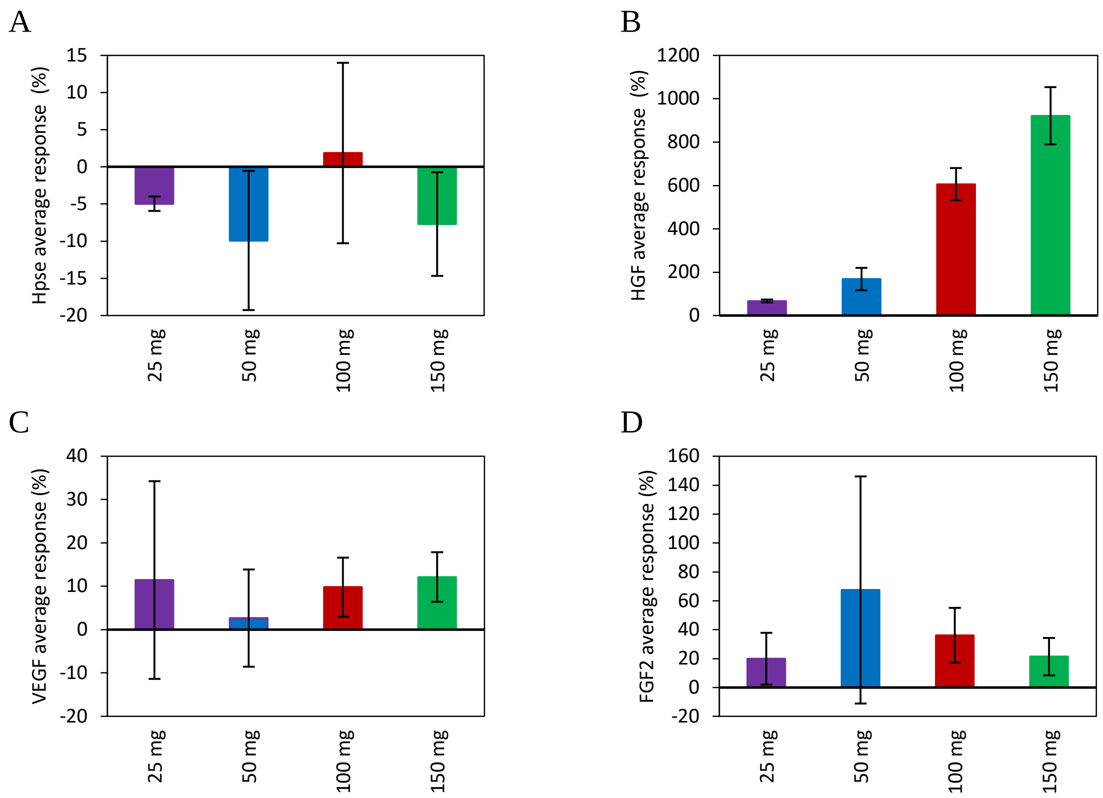

Figure S4. Modulation of extracellular matrix proteins measured in patient plasma during PG545 treatment. Average change in the analyte concentrations measured during treatment compared to the pre-dose level was expressed as a percentage increase or decrease. Data for heparanase (A), HGF (B), VEGF (C) and FGF2 (D) are plotted for each cohort with standard errors as error bars.

Table S3. Summary of observed modulations in immune markers are summarised for the 50 and 100 mg cohorts. Where increases greater than 2 fold were observed in these markers, they are indicated in green. Data plotted in Figure 3.

		pDC		NK cells		Plasma proteins			
		CD40$^+$	IFNα$^+$	CD335$^+$	IFNγ$^+$	IFNγ	TNFα	IP-10	MCP-1
50 mg	3021				green		green		
	2022						green		
	3023								
	3024	green	green	green	green				
	2025				green				
100 mg	1041		green	green			green		
	3042								
	3043			green		green	green	green	green
	3044								
	1045	green	green			green	green		green
	3046								green

A

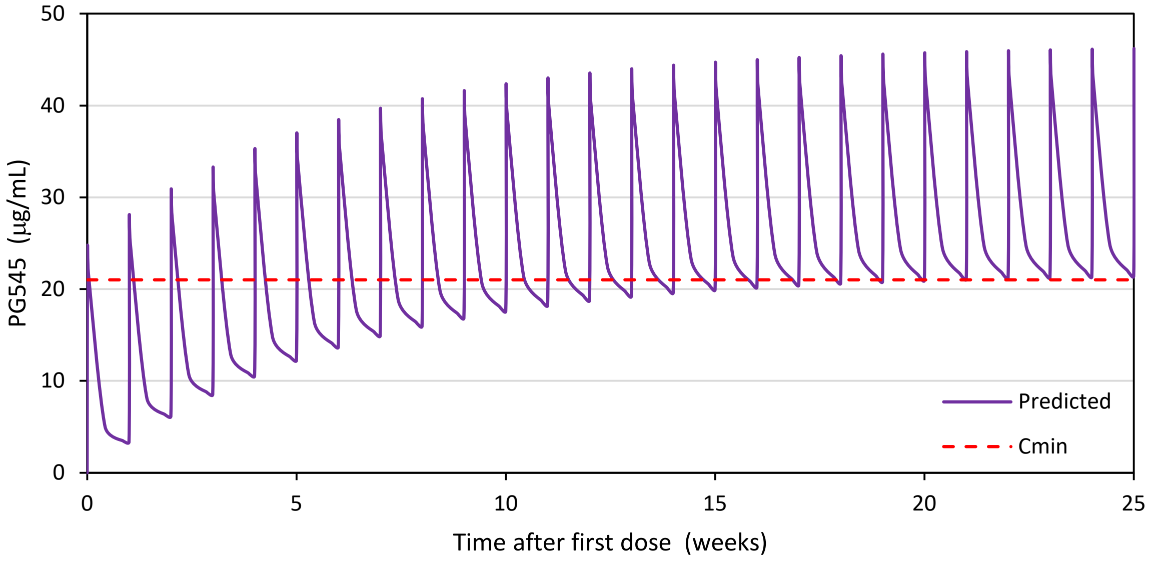

B

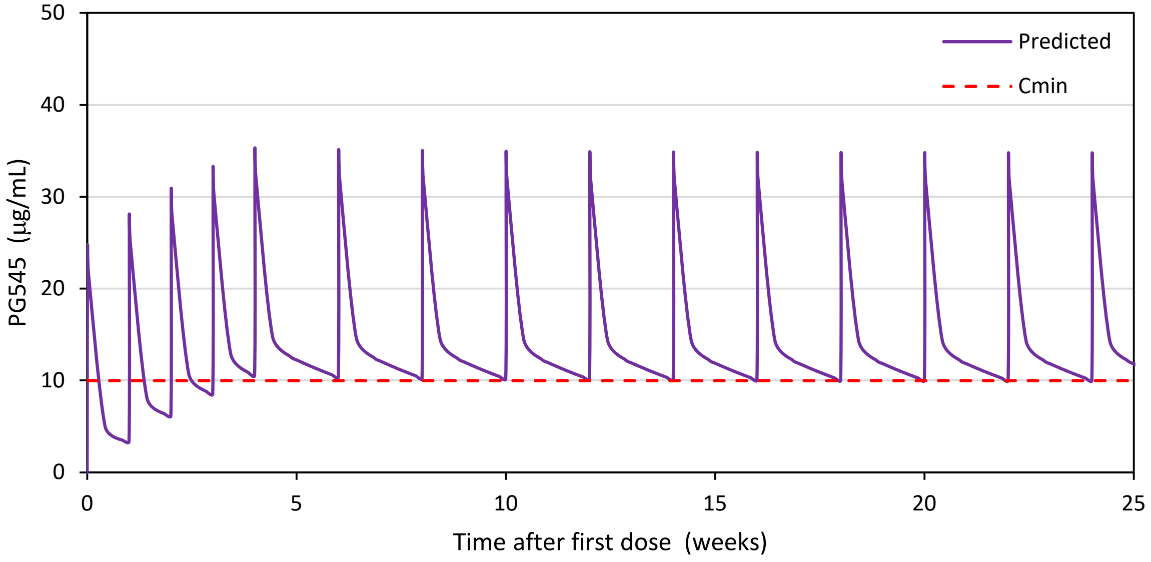

Figure S5. Simulated population mean PG545 concentrations predicted by PK model. Simulated dosing was either, once weekly at 100 mg (A), or once weekly at 100 mg for 4 weeks followed by once every two weeks (B). Predicted PG545 mean population plasma concentrations are shown by the purple lines and the steady state predicted C_{min} concentration for each dosing regimen are indicated with red dashed lines.
